# Recent advances in biomarker detection of oral squamous cell carcinoma

**DOI:** 10.3389/fonc.2025.1597086

**Published:** 2025-06-19

**Authors:** Lan Liu, Xiaowu Zhong, Yue Zhong, Lihua Li

**Affiliations:** ^1^ Department of Stomatology, Affiliated Hospital of North Sichuan Medical College, Nanchong, China; ^2^ Department of Clinical Laboratory, Affiliated Hospital of North Sichuan Medical College, Nanchong, China

**Keywords:** oral squamous cell carcinoma, single-cell sequencing technology, spatial transcriptomics, nanopore sequencing, artificial intelligence technology, biosensor technology

## Abstract

Oral squamous cell carcinoma is among the most prevalent tumours of the oral and maxillofacial region. The initial symptoms are typically minor and may remain misdiagnosed until the disease advances, resulting in a significantly reduced five-year survival rate for patients. Early detection is critical, as it can improve five-year survival rates from below 50% to 70–90%. Due to their reduced sensitivity and intrusive nature, conventional screening methods such as serological testing and histopathological biopsies have limitations in their application. In contrast, emerging technologies including single-cell sequencing, spatial transcriptomics, nanopore sequencing, biosensor technology, and artificial intelligence, among other advanced detection methods, are redefining biomarker discovery. Scalability obstacles still exist, including clinical validation gaps, high implementation costs, and analytical complexity. In order to close the gap between invention and equitable implementation, future efforts should focus on multicenter validation of potential biomarkers and cost-effective integration of these technologies. This will ultimately improve patient prognosis and quality of life. This work aims to comprehensively investigate and evaluate the prospective applications and future developmental potential of these technologies while offering an extensive examination of oral squamous cell cancer biomarker research

## Introduction

Oral squamous cell carcinoma (OSCC) is the most prevalent malignancy of the oral cavity, accounting for over 90% of all oral cancers (1), with an estimated 389,000 new cases and 188,000 deaths annually globally each year (2). Significantly, incidence rates are increasing threefold in low- and middle-income nations (LMICs) relative to high-income regions (3), highlighting differences in healthcare access and delayed diagnosis. Early symptoms (e.g., painless ulcers or erythroplakia) are frequently overlooked, leading to diagnosis at advanced stages (T3/T4) in 60-70% of LMIC patients versus 30-40% in high-resource settings (4). This disparity stems from systemic barriers, including the high cost of molecular tests ($200–500 per analysis) (5), even exceeding monthly incomes in many LMICs, and their specialized oncology centers are concentrated in urban areas (6). Late diagnosis also reduces treatment efficacy; the 5-year survival rate plummets from 84% for localized tumors to 39% for metastatic disease (7). And even after treatment, extensive surgeries frequently cause permanent functional impairments, including speech deficits (45% of patients) and feeding tube dependence (32%) (6). These data underscore the urgent need for accessible early detection tools that can overcome geographic and economic divides.

**Figure 1:**
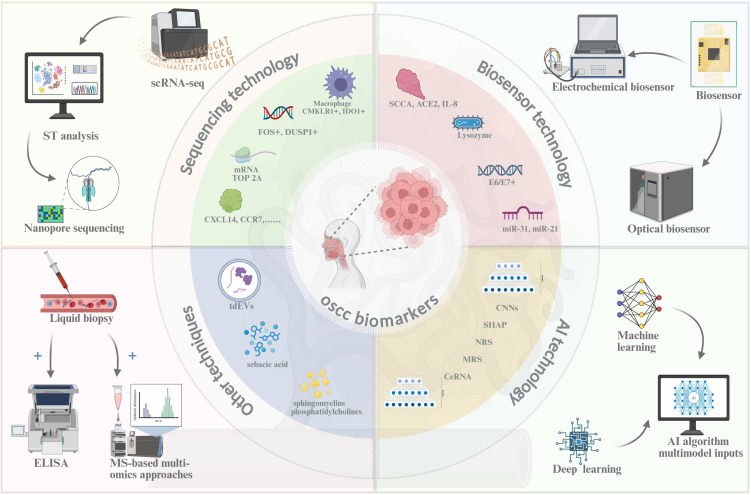
New detection technique of OSCC. Created in https://BioRender.com.

Traditional diagnostic techniques for OSCC, such as direct inspection, histopathological analysis, chemical staining, and exfoliative cytology, remain the clinical standard. However, their invasive nature (e.g., requiring tissue biopsies), low sensitivity (60–75%) (4), high costs (5), and reliance on specialized pathology expertise hinder widespread adoption, particularly in low-resource settings where diagnostic delays are common (6). Recent advances in molecular biology and artificial intelligence (AI), particularly the appearance of deep learning-based image analysis (AUC=0.87 for OSCC detection) (8), have transformed OSCC biomarker discovery and early detection (**Figure 1**). Emerging technologies including single-cell RNA sequencing (scRNA-seq, profiling 20,000 cells per run) (9), spatial transcriptomics (10-μm resolution) (10), biosensors (95% sensitivity for salivary miRNAs) (11), and AI (different algorithms can analyze large amounts of data), have identified novel biomarkers like OSCC proliferation (e.g., TOP2A mRNA) (12) and immune evasion (e.g., Galectin-9+ TAMs) (13), as summarized in **Table 1** ^1^. These innovations provide minimally invasive alternatives for early OSCC detection, yet their clinical translation requires overcoming cost barriers and validation in diverse populations (55). This study critically evaluates recent advancements in OSCC biomarker detection, emphasizing technological innovation and barriers to equitable implementation. We assess the translational potential of these tools while advocating for cost-reduction strategies and multicenter validation to bridge healthcare disparities. 

**Table 1:** Biomakers of oral squamous cell carcinoma.

Methods	Type	Sources	Potential biomakers	Verification phase	Expression	Effect value	Potential role	(Author, year)
scRNA- seq	Macrophage	Data cohort	CMKLR1+ macrophage	In vitro validation	upregulated	Not memtioned Related to better OS and RFS	Inhibition tumor progression	(Lou et al., 2024) (14)
		Tissues	Macro-IDO1	In vivo validation in mice	upregulated	Not metioned Positively related to CD8-exhausted	Immunosuppression and Promote tumor progression	(Y. Zhang et al., 2024) (15)
	Gene	PTs	RGS4+ mCAF1	In vitro validation	upregulated	Not metioned, related to prognosis	Immunomodulation and tumor microenvironment Modeling	(Q. Zhang et al., 2024) (16)
		LNs	COMP+mCAF2	In vitro validation	upregulated		Promote tumor progression	
		Tissues	FOS+, ATP1A+ , DUSP1+	In vitro validation	Correlation	Not metioned	Correlated with progression; Prognostic biomarker	(Kurkalang et al., 2023) (17)
			AEG-1+	In vivo validation in mice	upregulated	Related to worse OS and DFS	Promote tumor progression; Prognostic biomarker	(Yao et al., 2024) (18)
		Mouse models	CCR7+	Bioinformatics analysis	Correlation	Not metioned	Correlated with immune cell infiltration	(Z. Wang et al., 2024) (19)
		mCAFs	FN1+, CALD1+, SPARC+, TMSB10+,MT-CO3+,S100A6+,TIMP1+, FTH1+ , COL6A2+, LGALS1+	Bioinformatics analysis	upregulated	Not metioned	Correlated with tumor progression	(Wu et al., 2023) (20)
scRNA- seq	mRNA	Tissues	TOP2A mRNA	In vitro validation	upregulated	SMD =1.51 AUC = 0.96	Promote tumor progression; Diagnostic biomar	(Cheng et al., 2024) (12)
	Protein	CAFs	TGF-β1	In vitro validation	upregulated	86.4 pg/mL in CAF-CM vs 42.8 pg/mL in NF-CM	Promote tumor invasion and induced EMT	(Yang et al., 2022) (21)
			NNMT	In vivo validation in mice	upregulated	Not mentioned, Positively related to MVD	Promote tumor angiogenesis	(X. Wang et al., 2024) (22)
		Tissues	PLIN2	In vitro validation	upregulated	Higher expression in T3/T4 (p = 0.002)	Promote tumor progression; Related to prognosis	(He et al., 2022) (23)
			CCR7	In vivo validation in mice	upregulated	Not metioned	Impair immune response	(Yan et al., 2024) (24)
			TOX	In vitro validation	upregulated	Positively related to expression of PD-1 (r=0.2820, P < 0.0001)	Promotes T cell depletion and immunosuppression; Immunotherapy target	(J.Chen et al., 2021) (25)
			ZNF71	In vitro validation	downregulated	SMD = −0.52 95%CI=−0.95-−0.10	Reduce infection of HSV1 and accelerate cell cycle	(F-C. Jiang et al., 2022) (26)
			CXCL14	In vivo validation in mice	downregulated	Positively related to quantity of the TIL (r=0.93,p=0.0003)	Promote TIL infiltration and Inhibition tumor progression	(Parikh et al., 2020) (27)
scRNA- seq	Protein	Oral cells	ENO2	In vitro validation	upregulated	Not metioned	Promote tumor progression; related to worse progosis	(Miao et al., 2024) (28)
			CXCL12	In vitro validation	downregulated	Negatively related to the TNM (p = 0.021)	Promote tumor progression; related to worse progosis	(Yorozu et al., 2023) (29)
ST and scRNA- seq	Protein	Tissues	RGS5, SLC16A1	In vitro validation	upregulated	Not metioned	Promote tumor progression	(Z.Liu et al., 2024) (30)
			NCBP2	In vitro validation	upregulated	Higher expression in OSCC p < 0.05	Promote tumor progression	(X. Xu et al., 2023) (31)
			EIF4E3	In vitro validation	downregulated	Lower expression in OSCC p < 0.05	Inhibition tumor progression	
		CAFs	Galectin-9, MHC-I	In vivo validation in mice	upregulated	Not metioned	Restricted CD8+ T cells anti-tumor immune response	(C. Li et al., 2024) (13)
			PDGFRA, HIF1A	In vitro validation	upregulated	Not metioned	Participate in regulating immunosuppression	(Z. Liu et al., 2024) (30)
ST	Macrophage	Tissues	TREM2+ Multinucleated Giant Macrophages	In vitro validation	upregulated	Related to better prognosis	Inhibition tumor progression	(Gessain et al., 2024) (32)
	Gene	Tissues	COL5A1	In vitro validation	upregulated	Not metioned	Promote tumor matastasis	(Shaikh et al., 2024) (33)
	Protein	Tissues	FN1	In vitro validation	upregulated	Not metioned	Promote tumor matastasis by remodeling of ECM	(Shaikh et al., 2024) (33)
			CXCL13	In vivo validation	upregulated	OSCC vs OLK 72.8 vs 33.5 pg/mL	Promote tumor progression and matastasis	(Tojo et al., 2024) (34)
Nano pore- seq	lncRNAs	Tissues	EPB41L4A-AS1, PSMA3-AS1, SLX1B-SULT1A4	In vitro validation	upregulated	Not metioned	Correlated with tumor progression	(Lin et al., 2024) (35)
			NEAT1, SNHG3, GAS5	In vitro validation	downregulated			
	Protein		RPS27A, RPL8	In vitro validation	Correlation			
ONT	Prevotella	Saliva	Prevotella melaninogenica, Prevotella intermedia, Prevotella jejuni	Bioinformatics analysis	upregulated	Not mentioned	Carcinogenic potential Inflammatory mediator Unclear	(Mauceri et al., 2023) (36)
	lncRNAs	Oral cells	DANCR,GAS5, NEAT1, CCDC144NL-AS1, SNHG3	Bioinformatics analysis	Correlation	Not mentioned	Regulation of gene expression	(Weber et al., 2023) (37)
	Protein		RPL21	Bioinformatics analysis	upregulated	Not mentioned	Translation regulation	
Electrochemical biosensor	Gene	Tissues	E6/E7+ on HPV-16 and HPV-18	In vitro validation	upregulated	Not mentioned LOD:22fM and 20fM	Carcinogenic potential; Diagnostic biomarker	(T et al., 2022) (38)
	Protein	OEC- M1s	ACE2	In vitro validation	Correlation	LOD: 0.1μg/mL	Key receptors for SARS-CoV-2 infection	(Lv et al., 2021) (39)
Electrochemical biosensor	Protein	Oral cells	SCCA	In vitro validation	Correlation	LOD: 0.01 pg/mL 0.05 pg/mL - 20.0 ng/mL	Diagnostic biomarker	(Y. Jiang et al., 2023) (40)
		Cell lines	MGMT	Bioinformatics analysis	downregulated	LOD: 0.24 × 10^-12^mol/L	Hypermethylation is closely associated with tumorigenesis; Diagnostic biomarker	(Carr et al., 2020) (41)
		Saliva	IL-8	In vitro validation	upregulated	LOD: 0.0001 ng/mL 0.004 - 10 ng/mL	Diagnostic biomarker	(Bhardwaj et al., 2024) (42)
Optical biosensor	Protein	Saliva	IL-8	In vitro validation	upregulated	LOD: 0.91 fM Sensitivity: 273 aM - 100 nM	Diagnostic biomarker	(Rashidova et al., 2024) (43)
SERS	Bacter-ia	Saliva	Lysozyme	In vitro validation	upregulated	Not mentioned	Correlated with progression; Diagnostic biomarker	(Farnesi et al., 2023) (44)
	ctDNA	Serum	TP53, PIK3CAE545K	In vitro validation	upregulated	Sensitivity: 100 aM - 1 nM	Diagnostic biomarker	(G. Li et al., 2022) (45)
	Mi- RNA	Saliva	miR-31, miR-21	In vitro validation	upregulated	Sensitivity: 10 aM - 10 pM	Diagnostic biomarker	(Y. Wang et al., 2022) (46)
AI	Chromosome	Tissues	chromosome 9p loss prediction, 9PLP	Bioinformatics analysis	Loss	AUC = 0.825	Immune microenvironment changes and immune escape	(Cai et al., 2024) (8)
	Gene	HPV- Tissues	ECT2	Bioinformatics analysis	Correlation	Not mentioned	Promote tumor progression	(Sekaran et al., 2024) (47)
	DEmRNA	Tissues	AC024592.9, LINC00941, LINC01615, MIR9-3HG	Bioinformatics analysis	correlation	Sensitivity = 96.2% AUC = 0.983 (SVM)	Promote tumor progression	(Hu et al., 2020) (48)
	snoRNA	Tissues	SNORD114-17, SNORD78, U3 (chr2)	Bioinformatics analysis	upregulated	AUC=0.674, 0.704, 0.66 (1,3,5-year survival rate)	Correlated with the malignant phenotype of the tumor	(Xing et al., 2020) (49)
			SNORA36B and U3 (chr17)	Bioinformatics analysis	downregulated		Protective biomarker	
	Protein	Oral cells	nuclear F-actin	Bioinformatics analysis	upregulated	OR = 1.97	Diagnostic biomarker; Related to early stage	(McRae et al., 2021) (50)
		Tissues	CDKN2A	Bioinformatics analysis	upregulated	AUC = 0.710 (Validation Set)	Cell Cycle Regulation and Tumor Suppression; Prognostic biomarker	(Y. Wang, Zhou, et al., 2024) (51)
			Ki-67 nuclear antigen	Bioinformatics analysis	upregulated	OR = 3.715 (T3/T4) CI: 1.580–8.737 P=0.003	Promote cell proliferation and inhibit apoptosis; Prognostic biomarker	(Chen et al., 2024) (52)
			TREM2	Bioinformatics analysis	Correlation	HR< 1 Related to better OS	Prognostic biomarker	(Gessain et al., 2024) (32)
	Methylation marker	Tissues	GPR15, GNG12, GDNF	Bioinformatics analysis	upregulated	AUROC = 0.67 (Internal Validation Set)	Promote tumor progression	(Viet et al., 2024) (53)
	Expression marker	Tissues	IGHA2, SCG5, RPL3L, NTRK1, CD96, BMP6, TFPI2, EFEMP2, RYR3, DMTN, GPD2, BAALC, FMO3	Bioinformatics analysis	correlation	Correlated with year survival rate, c-statistic = 0.9409	Prognostic biomarker	(Viet et al., 2024) (53)
	MRGs	Tumer samples	Macrophage-related risk signature, MRS models: IGF2BP2, KRT9, PPP1R14C, SLC7A5, RAC2, NTN4, CTLA4, APOC1, CYP27A1	Bioinformatics analysis	Correlation	Not mentioned	Predicting Tumor Prognosis and Response to Immunotherapy; Prognostic biomarker	(Y. Wang, Mou, et al., 2024) (54)

scRNA-seq, single cell RNA sequencing; OS, Overall Survival; RFS, Recurrence free survival; DFS, Disease-free survival; PTs, primary tumors; LNs, lymphatic node; mCAFs, matrix cancer-associated fibroblasts; CAFs, cancer-associated fibroblasts; CI, Confidence Interval; SMD, Standardized Mean Difference; AUC, Area Under the Curve; CAF-CM, Cance-Associated Fibroblast-Conditioned Medium; NF-CM, Norml Fibroblast-Conditioned Medium; EMT, Epithelial-Mesenchymal-Transition; MVD, Microvessel Density; TNM, Tumor, Node, Metastasis; TIL, Tumor-Infiltrating-Lymphocytes; ST, spatial Transcriptomics; lncRNAs, long non-coding RNAs; ONT, Oxford Nanopore Technology; LOD, Limit of Detection; OEC-M1s, human oral squamous cell carcinoma cell line 1; DEmRNA, differentially expressed messenger RNA; snoRNA, small nucleolar RNA; MRGs, Macrophage-related risk signature.

## Progress in research on new detection methods

### Sequencing technology

The scRNA-seq has transformed OSCC research by enabling high-resolution profiling of tumor heterogeneity (9) by analyzing gene expression at the individual cell level, with modern platforms (e.g., DNBSEQ-T20×2) achieving throughputs of 50,000 cells per run (14), while maintaining a low per-cell cost (~$0.01/cell) (56). It has identified candidate biomarkers including tumor-specific gene clusters, though further validation is needed to confirm their clinical utility (57). Combining scRNA-seq with single-cell regulatory network inference and clustering analysis (SCENIC) analysis identified CMKLR1+ macrophages (HR=2.1 for poor prognosis) (15), which may directly influence epithelial cell proliferation and impede OSCC progression. Typically, scRNA-seq directly quantifies mRNA expression in OSCC cells, revealing upregulated oncogenic transcripts such as TOP2A (3.2-fold increase in T3/T4 tumors) (12) and NNMT (correlating with smoking status, p<0.01) (22). However, TOP2A’s diagnostic specificity is limited by its expression in 18–22% of oral potentially malignant disorders (OPMDs) (12), necessitating complementary biomarkers (e.g., CXCL12, AUC=0.81) (29) to improve early detection accuracy. To enhance reliability, scRNA-seq findings can be cross-validated through orthogonal methods including immunohistochemistry, qPCR, and gene set enrichment analysis (GSEA), establishing a multi-platform verification framework. Beyond transcriptional profiling, scRNA-seq has facilitated the discovery of protein-coding biomarkers including the Immune modulators [CXCL12 (29) and CXCL14 (27)], Metabolic regulators (NNMT) (22), and [DUSP1 (17) and ZNF71 (26)], which were evaluated for gene expression of proteinaceous substances. The variations in the expression levels of these genes suggest their potential role as biomarkers for OSCC, but these markers still require prospective validation in multicenter trials before clinical implementation.

Spatial transcriptomics (ST) preserves tissue architecture while mapping gene expression, allowing researchers to study cellular interactions within tumor niches, bridges single-cell resolution with tissue context, preserving architectural details at 10-μm resolution while capturing >20,000 RNA molecules per spot (10), with next-generation platforms like Stereo-seq achieving 500 nm resolution (58). In OSCC, ST has uncovered tumor-zone-specific signaling gradients, such as 3.5-fold elevated WNT5A expression at invasion fronts (p<0.001) (59). Through the use of CellPhoneDB and NicheNet for intercellular communication analysis, ST technology has revealed the upregulation of HIF1A (4.2-fold in hypoxic niches, FDR<0.01), PDGFRA (2.8-fold in αSMA+ iCAFs), and RGS5 (1.9-fold in perivascular zones) (30) in the high metabolic region, indicating that iCAF transformation dictates the metabolic signature of the immunosuppressive microenvironment. Integrated analysis using scRNA-seq, immunohistochemistry, flow cytometry and other techniques, the Galectin-9 (92% of CD163+ TAMs, HR=2.1 for poor prognosis), MHC-I (68% tumor cells vs normal, correlates with CD8+ T cell exclusion), SLC16A1 (PET-CT SUVmax correlation r=0.79), and the chemokines CXCL9, CXCL10, and CXCL12 (13) were found to be upregulated in OSCC tissues or cells, and detect the upregulated expression of these genes may facilitate the early diagnosis. These discoveries enable the early detection that combined biomarker panels achieve 89% sensitivity (8), and to better achieve treatment stratification such as CXCL12 high tumors show a 3.2-fold better response to immunotherapy (60).

Oxford Nanopore Technology (ONT), a third-generation sequencing platform renowned for its real-time analysis capabilities, has demonstrated clinical utility in both pathogen detection (COVID-19 identification within 6 hours) (61), and cancer genomics (25). It can offer long-read-length sequencing and real-time detection via the electronic sequencing of raw DNA and RNA in OSCC detection. As the only commercially available direct RNA sequencing platform, ONT’s long-read capability resolves full-length lncRNA structures, revealing the Prognostic lncRNAs including DANCR isoforms (lymph node metastasis OR=3.2, 95%CI 1.8-5.7) and GAS5 variants (cisplatin resistance AUC=0.81) (37) and the microbial biomarkers including Prevotella spp. (detected in 68% OSCC cases vs 22% controls) (36), a prevalent aetiological factor in oral diseases intricately associated with the invasive and migratory characteristics of OSCC.

### Biosensor technology

Modern biosensors synergistically combine biorecognition elements (e.g., antibodies, aptamers) with advanced transducers, and convert molecular interactions into quantifiable optical/electrical signals within 10–15 minutes, enabling real-time monitoring of disease progression (11). Contemporary biosensor platforms are primarily categorized into two primary types based on the signal utilized: electrochemical sensors and optical sensors. Electrochemical impedance spectroscopy (EIS) enables real-time, label-free monitoring of biomolecular interactions with 0.1° phase resolution (62). By utilizing the characteristics of EIS, Lv et al. (39) developed a Pd@ACE2 nanosensor achieving 0.8 pM ACE2 detection in OSCC cells, single-cell analysis revealed ACE2 overexpression (3.2-fold) correlates with EMT markers (vimentin, E-cadherin), suggesting its role in OSCC metastasis. In addition, the EIS-based electrochemical sensor successfully identified the upregulation of IL-8 (2.4 ng/mL cutoff, 89% sensitivity for early OSCC) (42) and the downregulation of MGMT (83% specificity vs healthy controls) (41) in saliva, authentically achieved non-invasive sampling and cost-effective detection (48). Nanocomposite-based sensors enable breakthrough applications, gold nanoparticle/graphene nanosheet (Au/GN) complexes have been efficiently utilized as sensing electrochemical sandwich immunosensors, having proficiency in the detection of the squamous cell carcinoma antigen (SCCA) (40). Furthermore, a multi-analyte electrochemical gene sensor utilizing silicon nanoparticles (SiNPs) infused with various redox indicators can identify the E6/E7 genes of HPV-16 and HPV-18 (38), thereby offering novel opportunities for the concurrent detection of multiple biomarkers, and expand the repertoire of biomarkers for OSCC.

Optical sensors detect biomarkers that leverage light absorption, fluorescence, aggregation-induced luminescence (AIE), and light scattering, with several types of sensors available, such as colorimetric, fluorescent, and surface plasmon resonance (SPR) sensors. Recent material innovations have pushed detection limits to the single-molecule level. Cutting-edge applications include AuNP-based colorimetric arrays detecting miR-141 at 0.01 pM (Δλ=52 nm redshift) with 93% clinical accuracy for OSCC staging (63), SiQD-FRET systems quantifying GSH in 2 μL serum (0.1–100 mM range, R^2 = 0.99) for redox status monitoring (64), and the cost-effective and portable supersurface plasmon biosensor (MetaSPR), integrated with artificial nanoenzymes for use with the nanoenzyme-linked immunosorbent surface plasmon resonance biosensor (Nano-ELISPR) (65), achieving 0.01 pg/mL IL-6 detection in saliva within 8 minutes, priced at $0.50/test. Nano-ELISPR can undergo a reversible etching reaction through Ag ions on gold and silver MetaSPR chips, facilitating ultrasensitive and specific detection.

Surface-enhanced Raman scattering (SERS) leverages plasmonic nanostructures (e.g., Au/Ag nanoparticles) to achieve single-molecule detection sensitivity through localized surface plasmon resonance effects. It provides unique advantages including non-contact and non-destructive measurements, high resistance to interference, rapid data transmission, and telemetry control (66) for clinical translation, enabling simultaneous analysis of multiple targets in complex biological matrices. Integrated SERS platforms, which are combined with molecular dynamic (MD) simulation and analytical techniques, can achieve the IL-8 quantification [(2.4–100 ng/mL dynamic range)] (43) and lysozyme [(0.1-10 μg/mL)] detection (44) in saliva, as well as with lateral flow chromatography methods and catalytic hairpin assembly signal amplification strategies for the detection of circulating tumor DNA (ctDNA), such as TP53 (mutations at 0.01% allele frequency) and PIK3CA E545K in serum (45). Additionally, through hybridization chain reaction (HCR) amplification, SERS aims to identify diverse noncoding microRNAs, including miR-31 and miR-21 in saliva (46). Above all, SERS has emerged as a transformative OSCC diagnostic tool due to its sub-nm spectral resolution, single-cell sensitivity, and multiplex capacity while analysis, with 89% overall accuracy in a 500-patient cohort study (8).

### Artificial intelligence technology

Artificial intelligence (AI) systems emulate human cognitive processes to analyze complex biological data, demonstrating preliminary success in OSCC biomarker discovery but requiring rigorous clinical validation (53). Deep learning architectures, particularly convolutional neural networks (cNNs), have improved OSCC detection by analyzing histopathological images with 86.7% accuracy in margin assessment (53), though their performance varies across imaging modalities (67). Multi-omics integration through AI has revealed tobacco-associated epigenetic markers (GPR15, GNG12, and GDNF), these epigenetic alterations show a stronger correlation with tobacco exposure (p<0.001) than with tumor staging (p=0.12) based on multivariate analysis. In heavy smokers (≥10 pack-years), the mean methylation β-values increase by 0.38-0.45 compared to non-smokers. This finding supports targeted screening for high-risk populations. Moreover, Explainable AI (XAI) approaches have enhanced biomarker discovery in OSCC. Using Shapley Additive Explanations (SHAP) analysis combined with particle swarm optimization, researchers identified three prognostic biomarkers: ECT2, LAMC2, and DSG2 (47), and the downregulation of these genes signature correlates with poor clinical outcomes, and patients showing concurrent downregulation of all three markers exhibit a 3.2-fold higher metastasis risk (95% CI: 1.8-5.6) according to recent TCGA data analysis.

Machine learning (ML) models have demonstrated 75-89% cross-validation accuracy in analyzing OSCC gene expression profiles from TCGA datasets (68), are increasingly applied to analyze gene expression profiles in OSCC. These models quantify tumor heterogeneity through cellular diversity indices (e.g., Shannon entropy) and spatial patterning metrics, achieving 0.81 AUC for distinguishing early-stage lesions, though with variable performance across histological subtypes (54). AI-enhanced histopathology has revealed recurrent loss of heterozygosity (LOH) at chromosome 9p (38% frequency), implicating tumor suppressor genes such as CDKN2A (8). This finding suggests a potential role in early carcinogenesis, though functional validation is ongoing. Furthermore, Gradient boosting machine (GBM) models have identified CDKN2A inactivation in 72% of TCGA-analyzed OSCC cases, correlating with dysregulated G1/S checkpoint control (51). This positions CDKN2A as a high-priority biomarker candidate, pending multicenter validation. Multiparametric ML-MRI integration has demonstrated prognostic utility, with Ki-67 expression serving as an independent predictor of poor survival (HR=2.3, 95% CI 1.7–3.1) (52). However, its clinical adoption is limited by interobserver variability in immunohistochemical scoring. Advanced neural networks (e.g., SurvNet) integrated with explainable AI (XAI) frameworks have optimized the selection of multimodal biomarkers. Specifically, a recent multicenter study validated the prognostic value of combining p16 status (AUC=0.87), FDG-PET-derived MTV50 (HR=2.1), DCE-MRI blood volume (cut-off >12 mL/100g), and ADC values (<1.2×10–^3^ mm²/s) (55). These indicators are crucial for OSCC staging, evaluating treatment response, and assessing prognosis.

AI-driven multimodal systems demonstrate emerging potential for OSCC management, though their clinical implementation faces scalability challenges due to computational complexity and validation gaps (56). Through integrative analysis of histopathology, transcriptomics, and clinical data, these systems achieve 71-89% concordance with gold-standard diagnoses in controlled trials, yet real-world performance varies significantly across healthcare settings (69). AI-enhanced Raman imaging achieves 86.7% accuracy for intraoperative margin assessment in single-center studies, though multicenter validation is needed to confirm generalizability (67). Age-stratified AI models improve TNM staging prognostic value (AUC 0.65-0.72), with the most significant benefits observed in elderly cohorts where clinical judgment variability is highest (70). Nodal risk prediction models (NRS) combining radiomic features (e.g., DCE-MRI blood volume >12 mL/100g) and histomorphometric data achieve 82% accuracy for metastasis detection, surpassing conventional imaging by 15-20% (55). Network analysis-derived macrophage signatures (e.g., IGF2BP2, CTLA4) show 3.2-fold increased metastasis risk in high-risk subgroups but require prospective validation given potential overfitting in TCGA data (54). Validation in TCGA cohorts showed high-risk MRS patients had a 3.2-fold increased metastasis risk (p<0.001). Moreover, multiple ML algorithms have identified critical ceRNA networks involving HOXC13 and KLHL40, with TGFBR3 showing context-dependent roles. In elderly patients (≥65 years), HOXC13 downregulation correlates with advanced TNM stages (OR=3.22, p=0.002), while KLHL40 mutations are associated with smoking-related epigenetic changes (71). These integrated models (NRS, MRS, ceRNA) exemplify the convergence of multi-omics and clinical informatics in OSCC management and underscore the potential of AI to translate complex biomarker patterns into actionable clinical strategies.

### Other techniques

Beyond conventional histopathological methods, emerging multi-omics approaches have enhanced OSCC biomarker detection by integrating liquid biopsy, metabolomics, and lipidomic profiling. Liquid biopsy facilitates non-invasive OSCC monitoring by analyzing circulating tumor cells (CTCs), cell-free DNA (cfDNA), exosomes, and tumor-derived extracellular vesicles (tdEVs), with tdEVs exhibiting the highest sensitivity (AUC = 0.89) for early-stage detection (72). Dysregulated miRNAs, particularly miR-21 (upregulated in 78% of OSCC cases) and miR-31 (associated with lymph node metastasis) (73), serve as diagnostic biomarkers, whereas miR-200 family members (e.g., miR-200a/b/c) predict poor prognosis by promoting EMT (74). Mass spectrometry (MS)-based multi-omics approaches, particularly proteomics and lipidomics, have identified OSCC-specific metabolic alterations, such as aberrant glycolysis and fatty acid oxidation. Salivary metabolomics via GC–MS has revealed elevated sebacic acid levels (2.1-fold increase, p < 0.01) in OSCC patients compared to healthy controls, suggesting its potential as a non-invasive biomarker (75). In contrast, Lipidomic profiling further distinguishes OSCC by elevated sphingomyelins (SM d18:1/16:0, AUC = 0.91) and phosphatidylcholines (PC 34:1, AUC = 0.88), which correlate with tumor aggressiveness (76). Furthermore, an MS study integrated with lipidomics demonstrated that cholesterol and various phospholipids were markedly elevated in OSCC tissues (77), with machine learning models utilizing sphingolipid profiles achieving high diagnostic accuracy (AUC >0.95) (74).

### Challenges and prospects

Current OSCC biomarker detection methods face multifaceted challenges, including high costs (particularly for advanced sequencing technologies), inconsistencies in data interpretation due to heterogeneous sample processing protocols, difficulties in integrating emerging technologies with existing clinical workflows, and a lack of standardized validation frameworks, all of which hinder their widespread clinical adoption and result reproducibility. Despite advancements in sequencing technology enhancing the velocity of biomarker research, their reliance on complex bioinformatics pipelines for data analysis and substantial computational resource requirements disproportionately limit accessibility in resource-limited settings (56). Although Nanopore sequencing has demonstrated utility in pathogen surveillance due to its portability and real-time analysis capabilities, its application in early tumor detection requires substantial improvements in accuracy and cost-effectiveness (78). Emerging biosensor platforms, such as ELISA-based salivary biomarker detection systems, demonstrate high sensitivity for OSCC screening (e.g., detecting EGF: EGFR ratio changes with AUC >0.8) (79). However, their performance is influenced by pre-analytical variables including sample collection protocols, storage conditions, and ambient temperature fluctuations, mandating stringent standardization of operating procedures to ensure reliability (11). AI-driven approaches enhance diagnostic precision, although their clinical translation depends on overcoming challenges such as the scarcity of large-scale annotated datasets in oral oncology and the “black-box” nature of deep learning models, which complicates clinical validation and trust-building among practitioners (69). The transition from biomarker discovery to clinically actionable tools faces dual barriers. One is technical limitations in validating candidate biomarkers across diverse populations (e.g., single-center studies with limited sample sizes), and the other is regulatory and practical hurdles in implementing detection platforms within existing healthcare infrastructures. Above all, progress in this field demands innovative solutions for multimodal data harmonization (e.g., combining omics data with imaging and clinical records) and advanced computational frameworks.

Emerging innovations in OSCC biomarker detection are poised to revolutionize clinical practice, particularly through the integration of temporal-spatial multiomics and AI-enabled data synthesis. For instance, the Well-TEMP-seq platform enables dynamic tracking of gene expression changes at single-cell resolution during early carcinogenesis, providing critical insights into biomarker evolution (80). Recent breakthroughs in single-cell spatial transcriptomics, exemplified by STALocator (81) (integrating scRNA-seq with spatial transcriptomics for subcellular localization), have enabled high-resolution mapping of immune-stromal interactions within OSCC tumor niches. Nanotechnology-enabled biosensors are achieving unprecedented sensitivity thresholds with emerging applications in intraoperative margin assessment (67). AI-driven frameworks are overcoming data heterogeneity challenges through innovations like differentiable modeling architectures, offering novel insights for oral cancer biomarker identification. Additional technologies, such as ultrasensitive liquid biopsy assays, will further augment ctDNA and ctRNA detection, presenting a new avenue for non-invasive tumor identification. Interdisciplinary collaboration enhances technology integration, whereas governments and regulatory bodies must establish regulations to guarantee the safety and efficacy of developing technologies while facilitating their clinical implementation. In conclusion, technical innovation, interdisciplinary collaboration, and governmental endorsement will propel oral cancer biomarker detection into a new epoch of precision medicine, thereby improving patient survival and quality of life

## Footnotes

1 That listed biomarkers require multicenter validation before clinical adoption.
